# Changes in Rodent Abundance and Weather Conditions Potentially Drive Hemorrhagic Fever with Renal Syndrome Outbreaks in Xi’an, China, 2005–2012

**DOI:** 10.1371/journal.pntd.0003530

**Published:** 2015-03-30

**Authors:** Huai-Yu Tian, Peng-Bo Yu, Angela D. Luis, Peng Bi, Bernard Cazelles, Marko Laine, Shan-Qian Huang, Chao-Feng Ma, Sen Zhou, Jing Wei, Shen Li, Xiao-Ling Lu, Jian-Hui Qu, Jian-Hua Dong, Shi-Lu Tong, Jing-Jun Wang, Bryan Grenfell, Bing Xu

**Affiliations:** 1 State Key Laboratory of Remote Sensing Science, College of Global Change and Earth System Science, Beijing Normal University, Beijing, China; 2 Shaanxi Provincial Centre for Disease Control and Prevention, Xi’an, Shaanxi, China; 3 Department of Ecosystem and Conservation Sciences, University of Montana, Missoula, Montana, United States of America; 4 Department of Ecology and Evolutionary Biology, Princeton University, Princeton, New Jersey, United States of America; 5 Fogarty International Center, National Institutes of Health, Bethesda, Maryland, United States of America; 6 Discipline of Public Health, University of Adelaide, Adelaide, Australia; 7 UMMISCO, UMI 209 IRD—UPMC, 93142 Bondy, France; 8 Eco-Evolutionary Mathematic, IBENS UMR 8197, ENS, Paris, France; 9 Finnish Meteorological Institute, Helsinki, Finland; 10 Xi’an Centre for Disease Control and Prevention, Xi’an, Shaanxi, China; 11 Ministry of Education Key Laboratory for Earth System Modelling, Center for Earth System Science, Tsinghua University, Beijing, China; 12 Hu County Centre for Disease Control and Prevention of Shaanxi Province, Xi’an, Shaanxi, China; 13 School of Public Health and Institute of Health and Biomedical Innovation, Queensland University of Technology, Brisbane, Queensland, Australia; Santa Fe Institute, UNITED STATES

## Abstract

**Background:**

Increased risks for hemorrhagic fever with renal syndrome (HFRS) caused by Hantaan virus have been observed since 2005, in Xi’an, China. Despite increased vigilance and preparedness, HFRS outbreaks in 2010, 2011, and 2012 were larger than ever, with a total of 3,938 confirmed HFRS cases and 88 deaths in 2010 and 2011.

**Methods and Findings:**

Data on HFRS cases and weather were collected monthly from 2005 to 2012, along with active rodent monitoring. Wavelet analyses were performed to assess the temporal relationship between HFRS incidence, rodent density and climatic factors over the study period. Results showed that HFRS cases correlated to rodent density, rainfall, and temperature with 2, 3 and 4-month lags, respectively. Using a Bayesian time-series Poisson adjusted model, we fitted the HFRS outbreaks among humans for risk assessment in Xi’an. The best models included seasonality, autocorrelation, rodent density 2 months previously, and rainfall 2 to 3 months previously. Our models well reflected the epidemic characteristics by one step ahead prediction, out-of-sample.

**Conclusions:**

In addition to a strong seasonal pattern, HFRS incidence was correlated with rodent density and rainfall, indicating that they potentially drive the HFRS outbreaks. Future work should aim to determine the mechanism underlying the seasonal pattern and autocorrelation. However, this model can be useful in risk management to provide early warning of potential outbreaks of this disease.

## Author Summary

Hemorrhagic fever with renal syndrome (HFRS, caused by hantavirus) is a zoonotic infectious disease reservoired in rodent populations worldwide, but with 90% of the total cases occurring in China. Xi’an is one of the most endemic areas in China, with a total of 7,748 confirmed HFRS cases from 2005 to 2012. HFRS came to the attention of the public when two larger outbreaks occurred in Xi’an in 2010 and 2011, with 1,366 and 1,067 cases being reported, respectively. By using 8 years of surveillance data (2005–2012) on HFRS dynamics, including data on the main rodent host reservoir, human cases, and weather conditions, we show how the epidemic dynamics of HFRS were associated with seasonality, rodent abundance, rainfall, and temperature. We find that the two larger HFRS outbreaks coincided with the abrupt increase of rodent abundance and/or rainfall. We present a statistical model revealing strong effects of seasonality and autocorrelation and additional effects of rodent density and rainfall on HFRS incidence that gives robust prediction; this approach could be a very practical tool in Xi’an.

## Introduction

Hantaviruses (family Bunyaviridae, genus Hantavirus) are negative-stranded, trisegmented viruses that cause approximately 200,000 hospitalized cases annually, with case fatality rates of 0.5%–40%, depending on the virus [1,2]. In Eurasia, hemorrhagic fever with renal syndrome (HFRS), a rodent-borne viral disease caused by hantaviruses, is characterized by fever, hemorrhage, headache, back pain, abdominal pain, and acute renal failure and even death [3,4]. From 2006 to 2010, more than 50,000 HFRS cases in China were reported and therefore it remains an important public health issue in developing areas in China (mainly caused by two types of hantaviruses, Hantaan virus, HTNV; and Seoul virus, SEOV) [5–8]. Shaanxi Province is one of the most seriously affected areas in mainland China [5,9]. There were about 99,000 HFRS cases reported, and over 2,537 people have died from HFRS in Shaanxi Province in the last three decades.

Previous studies have revealed that climatic factors can influence HFRS transmission through their effects on the reservoir host (mostly rodents of the family Muridae) and environmental conditions [8,10]. Rainfall, in particular, is thought to affect rodent abundance through net primary productivity [11]. For example, rainfall can affect tree seed production which was found to be associated with outbreaks of rodent populations in deciduous forests [12,13]. Nephropathia epidemica (a type of HFRS) in Belgium was also shown to be preceded by abundant tree seed production [14]. In Southern China, positive correlations were observed between precipitation, absolute humidity, and annual HFRS cases; increases in rainfall were thought to increase the carrying capacity of the environment by increasing food availability, leading to increases in the rodent population and disease transmission [15]. Furthermore, rodent abundance was thought to influence HFRS transmission directly, through increased contacts between humans and rodents [16–18].

The ecology of rodent-borne hantaviruses is well-studied because of its threats to public health [19,20]. However, public spending on health and provision of technical assistance are insufficient in developing areas, leading to challenges in the control of HFRS and prevention of expansion and re-emergence. With its sudden onset and rapid progression, the case-fatality rate in untreated HFRS cases may reach up to 30%; with both morbidity and mortality occuring mainly in young adults, this disease can significantly affect the workforce and economy [21]. Misdiagnoses and delayed treatment, due to a lack of medical resources, may have contributed to the high mortality rate in the study area [22]. HFRS has been identified in all 31 Chinese provinces, and it is widely distributed in Mainland China. A risk assessment method with reliable prediction performance would provide early warning information for epidemics, and could assist in disease control and prevention via improving reservoir control and personal protection in developing areas.

**Fig 1:**
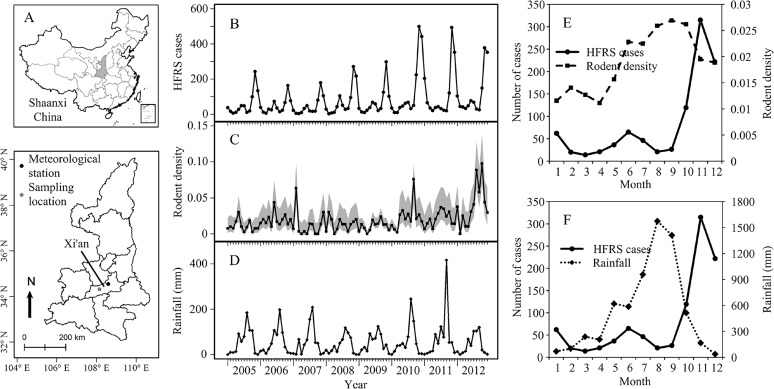
Epidemic pattern of HFRS in Xi’an, 2005–2012. (A) Sampling area in China. (B) Monthly distribution of HFRS cases. (C) The time series of rodent density; the grey areas indicate binomial 95% confidence intervals using the Agresti-Coull method, and (D) rainfall. Two major outbreaks were reported in 2010 and 2011. (D) Average seasonal distribution of HFRS cases and rodent density, 2005–2012. (E) Average seasonal distribution of HFRS cases and rainfall, 2005–2012.

Xi’an city is an area with a high incidence of HFRS in Shaanxi Province and a population about 8.46 million in 2010. Outbreaks have occurred every year since 2005, with varying magnitude, and the outbreaks in 2010 and 2011 were larger than ever, with 1,366 and 1,067 cases being reported, respectively (Fig. 1). This study investigated the association of HFRS outbreaks with climate variability and rodent abundance in Xi’an, China. We used the following framework to explore the predictive capability for HFRS epidemics. First, we performed wavelet coherency to examine the non-stationary association between variables and to compute the delays between HFRS cases and environmental and rodent variables. These two analyses helped form a set of plausible candidate models for prediction of HFRS incidence in Xi'an city. Second, these candidate time-series adjusted Poisson regression models were fit using Bayesian Markov chain Monte Carlo algorithms, with the best model selected using cross validation.

## Materials and Methods

### Background and data collection

Data on HFRS cases in Xi’an from 2005 to 2012 were obtained from the Shaanxi Notifiable Disease Surveillance System (HNDSS), which we were able to obtain in digital format in real time. All cases were first diagnosed according to the clinical criteria from the Ministry of Health of China, and blood samples were then collected from all suspected cases for serologic confirmation [23,24]. All sera from the patients were tested for specific IgM and IgG antibodies against hantavirus (including HTNV and SEOV). Serological and genetic analyses showed that all the cases were caused by HTNV.

Surveillance of rodent abundance in Xi’an from 2005 to 2012 was conducted once per month, for three consecutive nights, outside the town. The traps were placed 500 m away from villages in the fields (farmland or wasteland in Weihe Plain), which are the habitat for the important rodent reservoirs (according to the China National Surveillance Plan for HFRS control) using the following approach. A total of 100−1000 traps were set each night and were recovered in the morning. Traps baited with peanuts were placed outdoors in rows with 50 meters between consecutive rows, and every 5 meters along each row. In the field, each rodent was identified to species, killed with ether, and sent to the laboratory; detailed procedures can be found in published article [22]. Relative rodent density was calculated as the number of rodents captured divided by the number of traps set. A total of 729 rodents were captured out of 38,337 effective trap-nights.

The continuous daily records of climatic variables, including daily mean temperature, and rainfall from 2005 to 2012, were obtained from the local meteorological stations, and were used to calculate monthly average temperature, and monthly rainfall (Fig. 1).

### Ethical review

The present study was reviewed and approved by the research institutional review board of the Shaanxi Provincial Centre for Disease Control and Prevention. The review board determined that utilization of disease surveillance data did not require oversight by an ethics committee because only aggregated data were used in the data analysis and no personal information has been used. The Animal Ethics Committee of the Shaanxi CDC also waived approval for this study. Because the methods did not include animal experimentation, it was not necessary to obtain an animal ethics license. In addition, species captured in this study are not protected in China and none of the captured species are included in the China Species Red List.

### Wavelet time series analysis

We used wavelet analysis to explore the periodicity in HFRS cases, rodent density, and climate time series (S1 Fig.). The wavelet analysis can investigate and quantify the temporal evolution of the periodic components of time series [25,26]. We also conducted wavelet coherence analysis and phase analysis to quantify the non-stationary relationship between HFRS time series, rodent density, and climate variables. The wavelet coherence provides local information about where two nonstationary time series tend to oscillate simultaneously [15,27], e.g. whether the presence of a particular frequency at a given time in HFRS incidence corresponds to that of the same frequency at the same time in a climatic factor. Then phase analysis allows us to characterize the associations between time series, and to calculate the phase difference and evolution of the time lags for the seasonal component of the analyzed time series. The phase angle can be viewed as the rhythm of the time series, and the difference in the rhythm (the phase difference) between two time series can be converted into an instantaneous time lag. All significance levels were based on 1000 bootstrapped series [28]. All these analyses were performed with MATLAB software version 6.5 (MathWorks Inc., Natick, MA, USA).

### Cross-correlation analysis

The relationships between monthly incidence of the disease, climate variables, and rodent density were examined. Cross-correlation analysis was used to assess the associations, with consideration of lagged effects. To examine any lagged effects, lags of up to 6 months were included.

### Bayesian time-series adjusted Poisson model

Based on the results of the above analyses, we proposed a Bayesian time-series adjusted Poisson regression model to predict HFRS epidemics, which included autocorrelation, seasonality, and lagged effects of climatic variables (S1 File). To test the importance of these covariates, we also explored and ranked submodels and other biologically plausible candidate models. We used hierarchical Bayesian modeling with sampling-based methods for fitting. Here, we fitted the model by sampling the posterior distributions using Metropolis-Hastings Markov Chain Monte Carlo algorithm. Model fitting and model convergence (the convergence of numerical simulations [29]) were also done using MATLAB (vR2009b) toolbox DRAM (Delayed Rejection Adaptive Metropolis) [30,31]. We used five chains with different initial conditions to check for convergence of posterior distribution estimates. The prior distributions for the parameters were Gaussian, with a mean of 0 and a variance of 10^5^. An initial burn-in of 5,000 iterations was used, and posterior distributions of parameters were based on 5,000 more iterations. We only present the final results focusing on the median of posterior distributions and 95% credible intervals. We used a cross-validation approach that samples the first 80% of the dataset for fitting and the last 20% to test the model. The general model structure, used in the human HFRS epidemic analysis, was

$$Y_{t}∼\text{Poisson}\left(\mu_{t}\right)$$ (1)

$$\text{log}\left(\mu_{t}\right)=\alpha \;\text{log}\left(Y_{t-1}+1\right)+\beta X_{t}+\text{intercept}$$ (2)

where *Y*_t-1_ is the autocorrelation term, **X** is a vector of independent variables, potentially including rodent density, climatic variables, and seasonality. *β* is a vector of fixed-effects coefficients for the independent variables, including lagged effects. The best model was selected based on the pseudo-R^2^ and the Deviance Information Criterion (DIC). DIC is a measure of the fit of the model to the data that is penalized for the model’s complexity [32].

$$R^{2}=1-\frac{\sum_{i=1}^{N}{\left(y_{i}-{\hat{y}}_{i}\right)}^{2}}{\sum_{i=1}^{N}{\left(y_{i}-{\bar{y}}_{i}\right)}^{2}}$$ (3)

where *N* is the number of observations in the model, *y* is the dependent variable, $\bar{y}$ is the mean of the *y* values, and $\hat{y}$ is the value predicted by the model.

## Results

### Characteristics of HFRS epidemics

A total of 7,748 cases were confirmed in Xi’an between 2005 and 2012. The annual incidence of HFRS was 8.16/100,000 in 2005, 6.53/100,000 in 2006, 6.23/100,000 in 2007, 10.41/100,000 in 2008, and 9.58/100,000 in 2009, 17.81/100,000 in 2010, 16.98/100,000 in 2011, and 15.79/100,000 in 2012. The monthly distribution of HFRS cases indicated that HFRS incidence was higher in the second half of the year, from October to December (Fig. 1).

A total of 729 rodents were captured at monitoring sites in Xi’an. Captured rodents consisted mostly of the species *Apodemus agrarius*, *Rattus norvegicus*, and *Mus musculus*, which are known hosts of hantaviruses [33]. The capture rate was 1.90 per 100 trap-nights, and more than 80% of the captures were *A*. *gregarious*, the main reservoir of HTNV (Table 1). There was an annual peak of rodent density from August to October (Fig. 1).

**Table 1:** The number of rodents of each species captured, 2005–2012.

	*A*.*agrarius*	*R*.*norvegicus*	*M*.*musculus*	*C*. *barabensis*	Other species
**2005**	46	15	11	0	3
**2006**	30	3	3	0	6
**2007**	40	1	2	0	5
**2008**	85	20	17	0	6
**2009**	81	2	5	1	4
**2010**	56	8	6	0	2
**2011**	129	0	6	0	1
**2012**	129	4	1	0	1

### Correlations and wavelet coherences between climate variability, rodent density, and HFRS incidence

**Fig 2:**
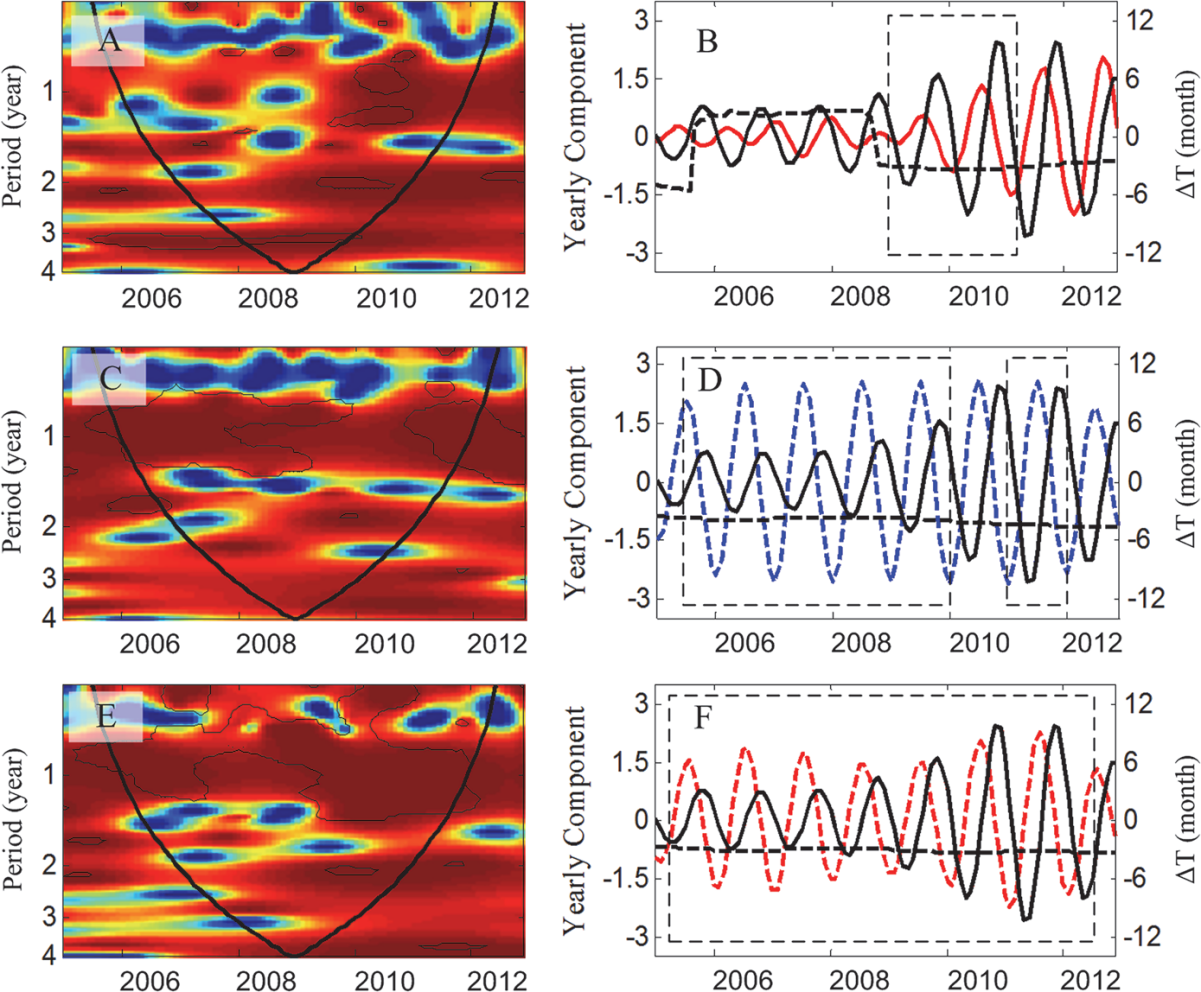
Association between climatic factors and the number of HFRS cases. **The incidence series are square root transformed, and all series are normalized.** (A) Association between rodent density and the number of HFRS cases by wavelet coherence; (B) Annual oscillating component (0.8–1.2 yr) evolutions of the considered series computed with the wavelet transform; the black thick line is HFRS cases, and the red line is rodent density. The coherences between HFRS cases and rodent density during 2005 to 2008, and after 2011 were not significant. (C) Association between temperature and the number of HFRS cases by wavelet coherence; (D) Annual oscillating component (0.8–1.2 yr) evolutions of the considered series computed with the wavelet transform; the blue dashed line is temperature. (E) Association between rainfall and the number of HFRS cases by wavelet coherence; (F) Annual oscillating component (0.8–1.2 yr) evolutions of the considered series computed with the wavelet transform; the red dashed line is rainfall. For A, C, and E, the coherence power spectra (x-axis: time in year; y-axis: period in year); power is coded from low value, in dark blue, to high value, in dark red. The black dashed lines show 5% significance level, computed on 1,000 bootstrapped series. This was used to quantify the statistical significance of the computed patterns, by constructing control datasets from observed time series that share properties with the original series, and comparing them with the original values computed from the raw series under the null hypothesis [28]. The inner area, within the cone of influence (black line), indicates the region not influenced by edge effects. For B, D, and F, black dashed boxes represent the period of time where coherency is significant in the 0.8–1.2-y period band, when interpretation of analysis was possible. Red line: rodent density; blue dashed line: temperature; red dashed line: rainfall; black lines: HFRS cases; dashed black lines: phase angle difference between the two oscillating components.

The correlation between HFRS incidence, the climate variables, and rodent density were calculated with a lag of 1–6 months in Table 2. The results indicated that monthly HFRS cases were positively correlated with rodent density with a 2-month lag (r = 0.38, *P* < 0.01). HFRS cases were preceded by rainfall and temperature with 3-month and 4-month lags, respectively. These results were consistent with the wavelet coherence analysis. Wavelet coherencies between the time series are shown in Fig. 2. Cross-wavelet coherence and phase showed that the dynamics of HFRS cases are associated with rainfall with a 3 month lag, temperature with a 4 month lag through 2009 and during 2011, and rodent density with a 2 month lag over the period 2009 and 2010 (Fig. 2).

**Table 2:** Cross-correlation coefficients of monthly variables and HFRS cases, 2005–2012.

Lag value	HFRS and rodent density	HFRS and rainfall	HFRS and temperature
Lag-1	0.26*	-0.02	-0.05
Lag-2	0.38*	0.49*	0.21
Lag-3	0.37*	0.65*	0.42*
Lag-4	0.36*	0.41*	0.53*
Lag-5	0.23*	0.13	0.48*
Lag-6	0.12	-0.03	0.3

### Bayesian model for the effect of environmental variables on HFRS outbreaks

Based on the results of wavelet coherence, correlations, and the clear seasonal pattern of observed HFRS cases (Fig. 1), our maximal Bayesian time-series adjusted Poisson regression model had the form,

$$\text{log}\left(\mu \right)=\beta_{1}\;\text{log}\left(Y_{t-1}+1\right)+\beta_{2}RD_{t-2}+\beta_{3}\;\text{log}\left(R_{t-3}+1\right)+\beta M+\text{C}$$ (4)

where *RD* denotes the relative rodent density, *R* denotes rainfall, and *M* is a seasonal dummy variable, denoting month, and *C* is an intercept term. Submodels and those exploring different time lags on the covariates (i.e., biologically meaningful and with acceptable model diagnostics) were also run and ranked based on fit by DIC and cross validation by pseudo-R^2^, see Table 3 below. Trace plots (S3 Fig.) and the Gelman and Rubin diagnostic indicated no lack of convergence.

**Table 3:** Model comparisons.

Variables	R-sq for prediction	DIC
Y(t-1), Rodent density(t-2), Rainfall(t-3), Season	0.82	135.80
Y(t-1), Rodent density(t-2), Rainfall(t-2), Season	0.81	124.78
Y(t-1), Rodent density(t-2), Rainfall(t-3), Temperature(t-4), Season	0.80	244.89
Y(t-1), Rainfall(t-3), Season	0.80	133.26
Y(t-1), Rodent density(t-1), Rainfall(t-3), Season	0.79	133.65
Y(t-1), Rodent density(t-2), Season	0.79	135.05
Y(t-1), Rodent density(t-1), Season	0.79	133.06
Y(t-1), Season	0.79	130.26
Y(t-1), Rodent density(t-2), Rainfall(t-1), Season	0.76	126.55
Rodent density(t-2), Season	0.69	285.08
Y(t-1), Rainfall(t-2)	0.66	466.97
Rodent density(t-2), Rainfall(t-3), Season	0.65	281.72
Y(t-1), Rodent density(t-1)	0.51	836.43
Y(t-1), Rodent density(t-2), Rainfall(t-3)	0.42	589.90
Y(t-1), Rainfall(t-1)	0.37	686.08

Our maximal model (Eqn 4) was the top model based on out-of-sample predictive power by pseudo-R^2^ (Table 3). The best model by in-sample fit (penalized for complexity) by DIC is a similar model, with the only difference being the time lag on rainfall—rainfall with a 2-month lag fit better than rainfall with a 3-month lag. These results indicate that autocorrelation, seasonality, relative rodent density 2 months previously and rainfall 2 to 3 months previously were associated with HFRS in Xi’an. The human HFRS cases were positively correlated to the relative rodent density, as well as with rainfall (Table 4). We found that adding temperature to the model with a lag of 4 months did not improve predictive power (Table 3).

**Table 4:** Posterior estimates, standard deviations (S.D.), and 95% credible intervals (CI) for the parameters.

Variables	Estimate	S.D.	95% CI
Lag-1 no. of cases, β1	0.97	0.13	0.73∼1.23
Lag-2 rodent density, β2	0.46	2.37	-4.19∼5.11
Lag-3 rainfall, β3	0.14	0.07	0.01∼0.28
Month-Jan	-0.73	0.24	-1.21∼-0.27
Month-Feb	-1.72	0.48	-2.67∼-0.79
Month-Mar	-0.10	0.74	-1.55∼1.35
Month-Apr	1.62	0.55	0.55∼2.71
Month-May	1.20	0.35	0.52∼1.90
Month-Jun	0.98	0.30	0.40∼1.58
Month-Jul	-0.033	0.25	-0.53∼0.45
Month-Aug	-0.46	0.35	-1.15∼0.23
Month-Sep	0.60	0.40	-0.17∼1.39
Month-Oct	1.45	0.32	0.83∼2.09
Month-Nov	1.08	0.17	0.76∼1.42
Intercept term	-0.79	0.75	-2.26∼0.68

**Fig 3:**
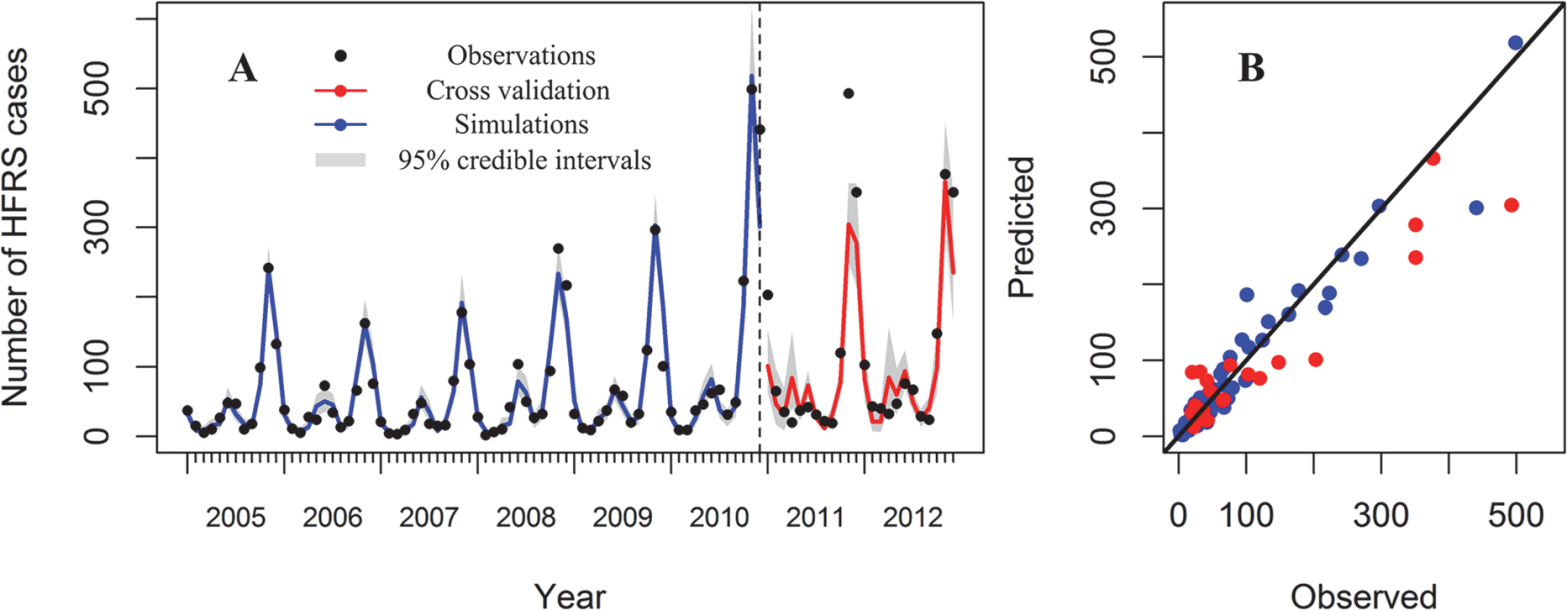
Observed versus simulated HFRS cases (One-step ahead prediction). The black points indicate observations; the blue indicates simulations from 2005 to 2010; the red indicates cross validation for 2011–2012. The grey areas indicate the 95% credible intervals of the model fit.

Using one-step ahead prediction (Fig. 3), the model fit the observed number of cases reasonably well over the period 2005–2012, including peak values; the pseudo-R^2^ value for the predicted model was 82.24%. As a diagnostic, we found that there was no significant autocorrelation in the residuals (S2 Fig.). Autocorrelation and seasonality account for much of the variation explained (pseudo-R^2^ value for the model including only a dummy variable for each month and the number of cases occurring in the previous month was 79.11; Table 3). Rodent density and rainfall have seasonal patterns and could in part explain the seasonality; however the model including these variables without an additional seasonal dummy variable only predicts 42% of the variation. This indicates that there are complex mechanisms in HFRS seasonal patterns still unexplained. Although the increase in predictive ability is small, the best model by DIC, which penalizes models for added complexity, includes rodents and rainfall, suggesting there may be a small but significant effect of interannual variability in rodent abundance and rainfall on HFRS incidence.

Multistep-ahead-predictions for the test dataset were also conducted. This will take the previously forecasted values into consideration to make the next step forecast. The results showed that predictions by 1–2 months ahead were acceptable; the predicted R^2^ values were 0.82 and 0.51, respectively. Because our predictions rely on the rodent density 2 months previously, forecasting more than 2 months ahead is more difficult.

## Discussion

This study investigated the association between HFRS outbreaks, environmental conditions, and rodent density. Correlation and wavelet analyses indicated that the climatic and rodent variables have lagged correlations with HFRS incidence. Based on these findings we proposed a set of Bayesian time-series Poisson adjusted models. The best models revealed strong effects of seasonality and autocorrelation and evidence for additional effects of rodent density with a 2-month lag and rainfall with a 2- to 3-month lag. These models predicted HFRS incidence one-month-ahead with pseudo-R^2^ values of 81–82%. These results are valuable since they point the way to an early warning signal prior to potential HFRS outbreaks via increases in rodent density or rainfall [15,34].

The effects of rodent density and rainfall on HFRS cases are illustrated by the larger than usual outbreaks that occurred in 2010, 2011, and 2012. The HFRS outbreak in 2010 was led by a large increase in rodent density and a slight increase in rainfall a few months earlier. The outbreak in October and November of 2011 was preceded by an extreme rainfall event in September (416 mm). The outbreak in 2012 was preceded by a large increase in rodent density. However, these apparent relationships are not driven by these 3 outbreaks alone. The Bayesian model was trained on the first 80% of the data and did not include the 2011 and 2012 outbreaks. Therefore, these patterns were present before that time.

Rodents are natural reservoirs of hantaviruses [2,34], however, one of the fundamental controversies until now is whether seasonal changes in rodent abundance can fully explain seasonal variation in HFRS cases [35], because the relationship between rodent abundance and the abundance of infectious animals is unclear [35]. The 2010 HFRS outbreak was associated with an abrupt increase in rodent abundance. This is most likely because high abundance may lead to more contact between humans and the rodent reservoir, and then increase risk of HFRS outbreaks. Moreover, increases in rodent density can lead to increases in the force of infection in the rodent population through density-dependent transmission, increasing the prevalence of infection in rodents [35,36]. Therefore, increased rodent population sizes may affect human infections not only through increased contact between humans and rodents, but also through increased transmission within the relevant reservoir populations. HFRS incidence appears to be affected by the population dynamics of the hantavirus rodent reservoir, which can be seen even without any specific data on pathogen dynamics in the host populations.

The potential effect of rainfall is illustrated by the events of 2011. During the second half of that year, increased rainfall occurred and closely coincided with the human HFRS outbreak. Rodent abundance and other climatic factors were not found to have any significant shifts at that time. Excessive rainfall and flooding can destroy rodent habitat, which can lead to rodent population diffusion and increase the possibility of contact between rodents and humans [15]. Moreover, harvest occurs in the study area starting from the end of September to October, during which time farmers may be more likely to be exposed to infected rodents, particularly during a strong rainfall event. High humidity is also known to increase virus survival in the *ex vivo* environment [37,38], which could increase the HFRS risk for humans.

Autocorrelation and seasonal factors were also important and accounted for much of the variability. With our use of a seasonal dummy variable, we did not explicitly examine seasonal mechanisms, but rodent abundance and rainfall exhibit seasonal patterns and may in part explain the seasonal pattern of HFRS cases. Since models that included rodents and rainfall performed better than purely seasonal models by both DIC and R^2^, this may suggest possible links between interannual variability in rainfall, rodent reservoir density, and human HFRS. Proving these causal relationships will require further study using dynamic models and long-term observations.

The limitations of this study should also be acknowledged. Many factors can contribute to HFRS transmission. The two outbreaks detected in 2010 and 2011, could also be due to other factors, e.g. human activities and movement, or population immunity. Then, as a population-level study, the potential problem of ecological fallacy is always unavoidable. Rodents were removed from the study area (not live trap and release), which may alter the density by removing individuals and may increase immigration. Since only one major outbreak coincided with each of the covariates (rodent density and rainfall), caution should be exercised. In particular, further studies of longer time series and in other areas are needed to substantiate these findings. Finally, we may be missing important additional factors that play important roles in the HFRS transmission, such as the hantavirus infection prevalence in rodents. However, without considering additional factors our model fits the data well.

In conclusion, this study shows the links between climate, rodent reservoir dynamics, and dynamics of HFRS in Xi’an. We found that the two HFRS outbreaks in Xi’an coincided with two different factors, a rodent population explosion and a strong rainfall event, respectively. We also found a strong and repeated temporal pattern among climatic factors, rodent abundance, and HFRS in the study area. However, rodent density or rainfall may partly explain strong seasonality of HFRS transmission, but not completely. It is difficult to tease apart the exact seasonal mechanism from the available data. These findings may enhance predictive capacity for HFRS epidemics in Xi’an, giving us the opportunity to implement preparation and mitigation strategies such as heightening public awareness and controlling the abundance of rodent hosts to prevent an outbreak.

## Supporting Information

S1 Fig Wavelet power spectrum. (A) The wavelet power spectrum of the reported monthly number of HFRS cases by the date of symptom onset (square root transformed). (B) The wavelet power spectrum of rodent density. (C) The wavelet power spectrum of temperature. (D) The wavelet power spectrum of rainfall. The left panel illustrates the wavelet power spectrum for the different series (x-axis: time in year; y-axis: period in year). The power is coded from low values, in dark blue, to high values, in dark red. Statistically significant areas (threshold of 5% confidence interval) in wavelet power spectrum (left panels) are highlighted with a dashed line; the cone of influence (region not influenced by edge effects) is also indicated. Finally, the right panels show the mean spectrum (solid line) with its significant threshold value of 5% (dashed line).
(TIF)

S2 Fig Autocorrelation function plot of the residuals for the human HFRS model. (TIF)

S3 Fig Trace Plots of five chains for each of the parameters. (TIF)

S1 File Supplemental materials. (DOCX)
