# Supplementary figures and images for: Changes in Rodent Abundance and Weather Conditions Potentially Drive Hemorrhagic Fever with Renal Syndrome Outbreaks in Xi’an, China, 2005–2012
Source: PLoS Negl Trop Dis. 2015 Mar 30;9(3):e0003530. doi: 10.1371/journal.pntd.0003530 (PMC4378853; doi:10.1371/journal.pntd.0003530)

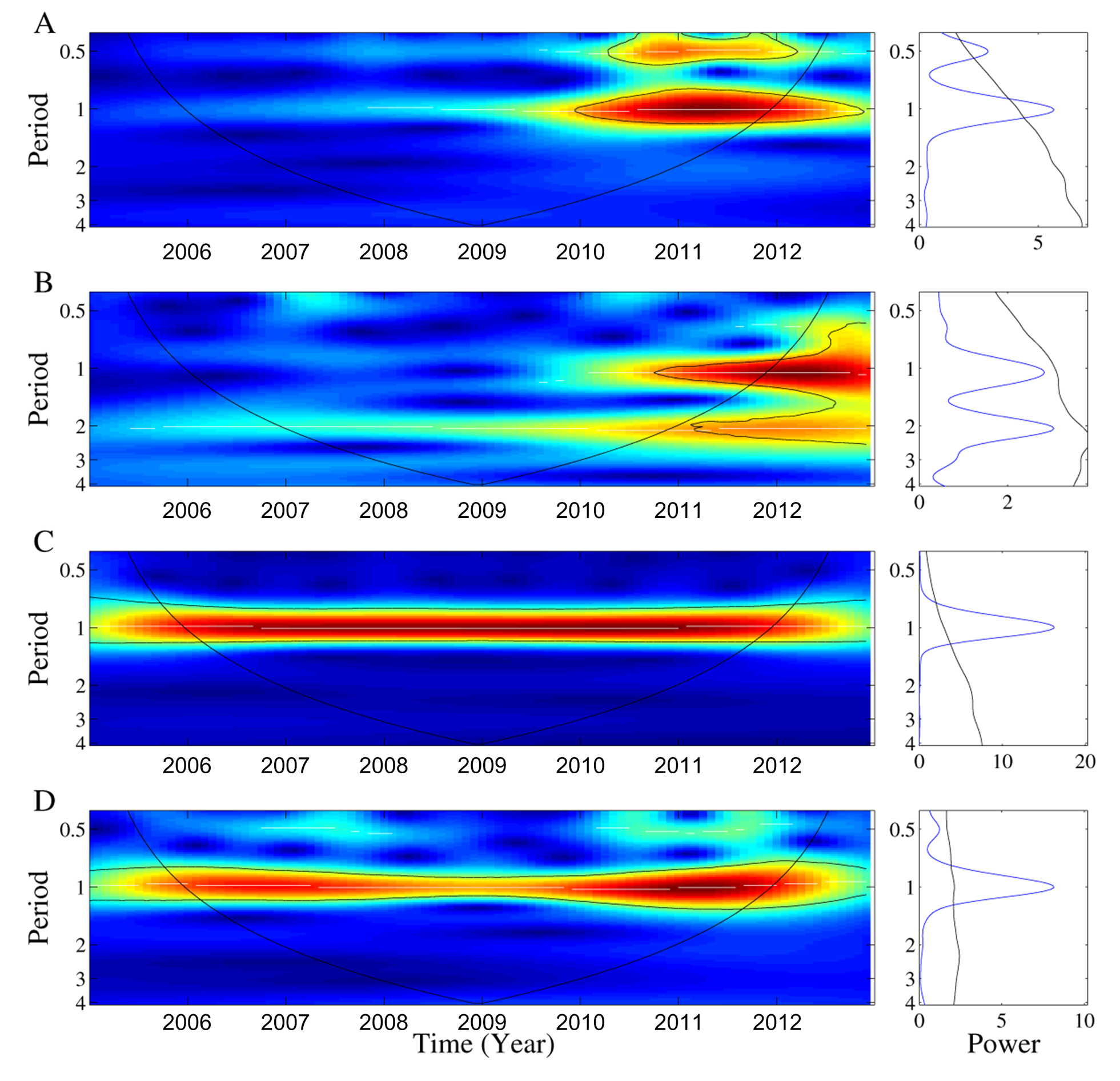

Supplement: S1 Fig — (A) The wavelet power spectrum of the reported monthly number of HFRS cases by the date of symptom onset (square root transformed). (B) The wavelet power spectrum of rodent density. (C) The wavelet power spectrum of temperature. (D) The wavelet power spectrum of rainfall. The left panel illustrates the wavelet power spectrum for the different series (x-axis: time in year; y-axis: period in year). The power is coded from low values, in dark blue, to high values, in dark red. Statistically significant areas (threshold of 5% confidence interval) in wavelet power spectrum (left panels) are highlighted with a dashed line; the cone of influence (region not influenced by edge effects) is also indicated. Finally, the right panels show the mean spectrum (solid line) with its significant threshold value of 5% (dashed line). (TIF) [file pntd.0003530.s001.tif]

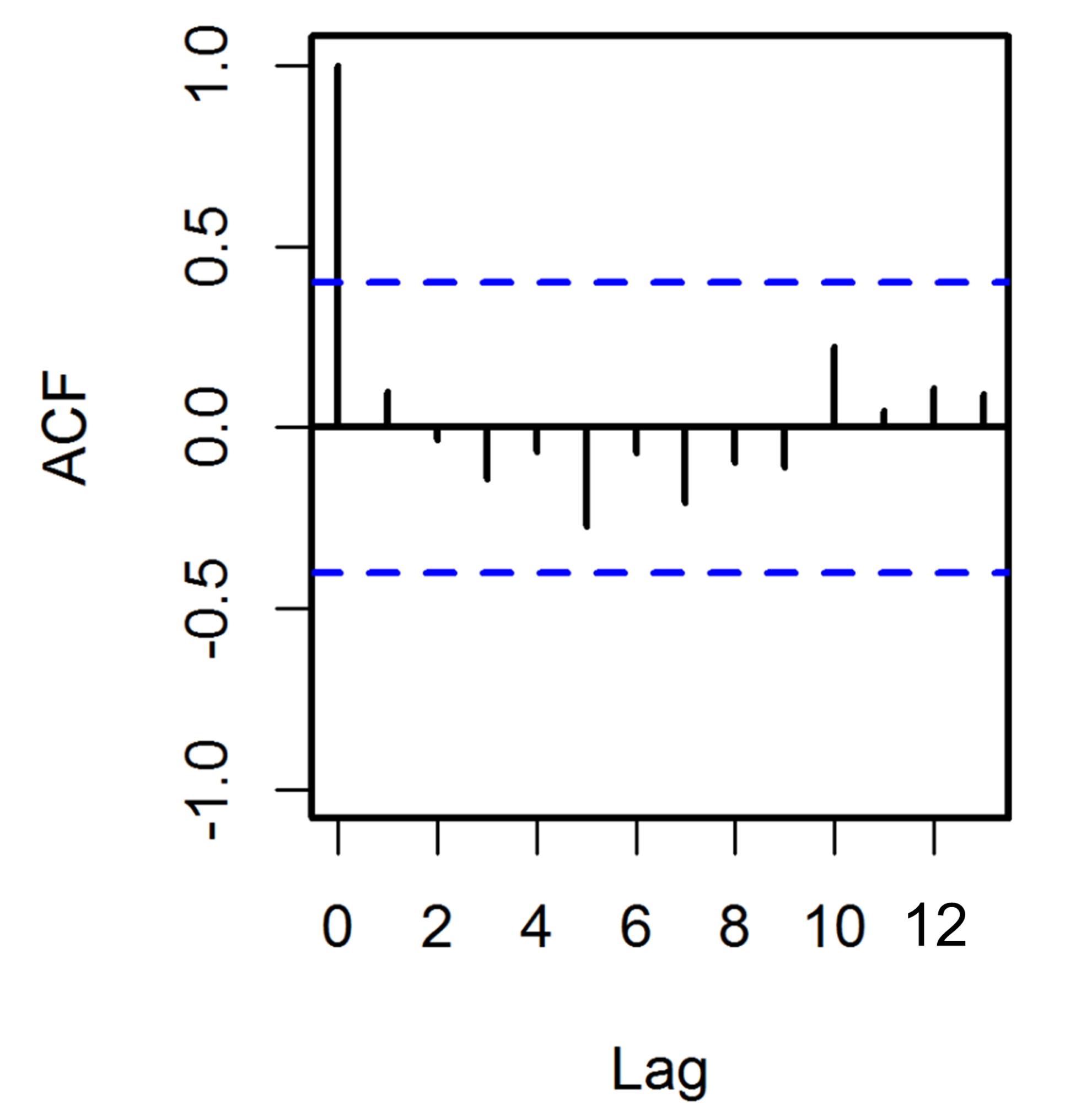

Supplement: S2 Fig — (TIF) [file pntd.0003530.s002.tif]

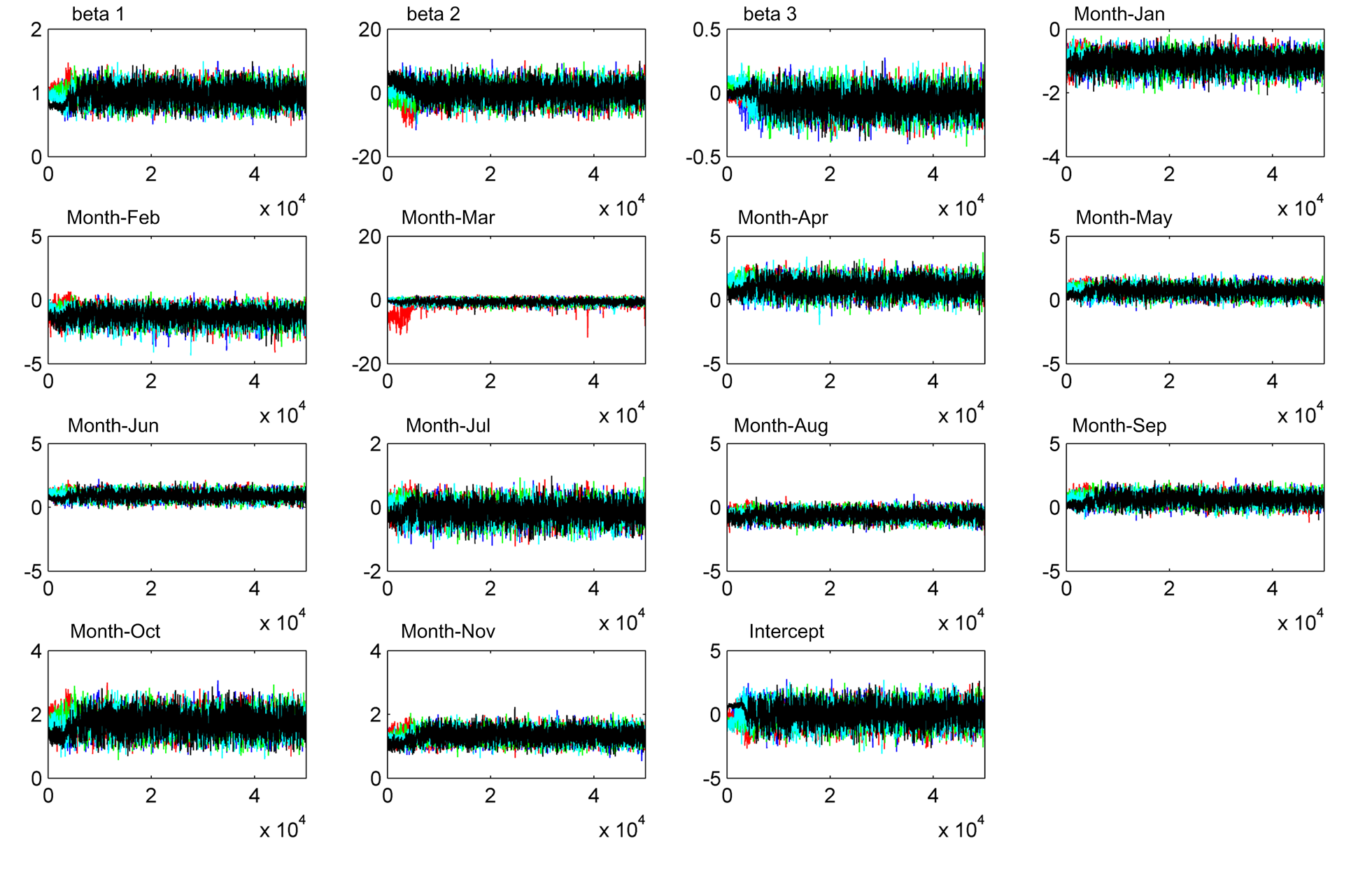

Supplement: S3 Fig — (TIF) [file pntd.0003530.s003.tif]
