# Supplementary material for: Changes in Rodent Abundance and Weather Conditions Potentially Drive Hemorrhagic Fever with Renal Syndrome Outbreaks in Xi’an, China, 2005–2012
Source: PLoS Negl Trop Dis. 2015 Mar 30;9(3):e0003530. doi: 10.1371/journal.pntd.0003530 (PMC4378853; doi:10.1371/journal.pntd.0003530)
Supplement: S1 File — (DOCX) [file pntd.0003530.s004.docx]

load train.mat

model.ssfun = @f3;

lo = -Inf;

params = {

{'beta1',0,lo,Inf}

{'beta2',0,lo,Inf}

{'beta3',0,lo,Inf}

{'beta4',0,lo,Inf}

{'beta5',0,lo,Inf}

{'beta6',0,lo,Inf}

{'beta7',0,lo,Inf}

{'beta8',0,lo,Inf}

{'beta9',0,lo,Inf}

{'beta10',0,lo,Inf}

{'beta11',0,lo,Inf}

{'beta12',0,lo,Inf}

{'beta13',0,lo,Inf}

{'beta14',0,lo,Inf}

{'beta15',0,lo,Inf}

};

model.S20 = [1];

model.N0 = [4];

% First generate an initial chain.

options.nsimu = 10000;

options.stats = 1;

[results, chain, s2chain,sschain]= mcmcrun(model,data,params,options);

% Then re-run starting from the results of the previous run,

options.nsimu = 50000;

options.stats = 1;

[results, chain, s2chain, sschain] = mcmcrun(model,data,params,options, results);

figure

mcmcplot(chain,[],results);

figure

mcmcplot(chain,[],results,'denspanel',2);

results.sstype = 1; % needed for mcmcpred and sqrt transformation

chainstats(chain,results)

modelfun = @(d,th) f2(d(:,1),th,d);

nsample = 500;

out = mcmcpred(results,chain,s2chain,data.xdata(1:end,:),modelfun,nsample);

figure

mcmcpredplot(out);

hold on

plot(data.ydata(1:end,2),'ks');

hold off

function y=f2(time,theta,xdata)

y = f4 (time,theta,xdata);

function ss = f3(theta,data)

time = data.ydata(:,1);

ydata = data.ydata(:,2);

xdata = data.xdata(:,:);

ymodel = f2(time,theta,xdata);

ss =sum((sqrt(ymodel) - sqrt(ydata)).^2);

function y = f4(t,theta,xdata)

x1= theta(1);x2= theta(2);x3= theta(3);x4= theta(4);

x5= theta(5);x6= theta(6);x7= theta(7);x8= theta(8);

x9= theta(9);x10= theta(10);x11= theta(11);x12= theta(12);

x13= theta(13);x14= theta(14);x15= theta(15);

Pre1=xdata(ceil(t),2);% Number of cases, lag-1 month

r=xdata(ceil(t),3);% rodent density, lag-0 month

r1=xdata(ceil(t),4);% rodent density, lag-1 month

r2=xdata(ceil(t),5);% rodent density, lag-2 month

rain1=xdata(ceil(t),6);%rainfall, lag-1 month

rain2=xdata(ceil(t),7);%rainfall, lag-2 month

rain3=xdata(ceil(t),8);%rainfall, lag-3 month

season=xdata(ceil(t),9);%season

s1=xdata(ceil(t),10);%Jan

s2=xdata(ceil(t),11);%Feb

s3=xdata(ceil(t),12);%Mar

s4=xdata(ceil(t),13);%Apr

s5=xdata(ceil(t),14);%May

s6=xdata(ceil(t),15);%Jun

s7=xdata(ceil(t),16);%Jul

s8=xdata(ceil(t),17);%Aug

s9=xdata(ceil(t),18);%Sep

s10=xdata(ceil(t),19);%Oct

s11=xdata(ceil(t),20);%Nov

Tem=xdata(ceil(t),22);%Temperature

I= exp(x1* log(Pre1+1) + x2*r2 + x3*log(rain3+1)...

+x4*s1+x5*s2+x6*s3+x7*s4+x8*s5+x9*s6+x10*s7+x11*s8+x12*s9+x13*s10+x14*s11 + x15);

y=I;
